# Litigation on kidney transplantation: 10-year experience from China

**DOI:** 10.1097/JS9.0000000000000625

**Published:** 2023-07-31

**Authors:** Qiyu He, Zhimin Tan, Liang Zhou

**Affiliations:** a Department of Urology; b Department of Anaesthesiology, West China Hospital of Sichuan University, Sichuan Province, Chengdu, People’s Republic of China

*Dear Editor*,

Kidney transplantation is the foremost treatment for end-stage renal disease^1^. China, ranking second globally in organ donation and transplantation, performs over 15 000 kidney transplant surgeries annually^2^. However, the rising number of medical accidents and disputes burdens healthcare providers and patients. Although general surgery-related litigation has been reviewed, there is a dearth of reports specifically addressing kidney transplantation litigation^3,4^. This study aims to review kidney transplantation-related litigation cases in China, exploring causes, medical responsibility determinations, and compensation outcomes. We hope the findings will provide education and guidance for healthcare professionals and ultimately enhance the prognosis of kidney transplant recipients.

Lexis China, launched by LexisNexis, is a comprehensive legal database in China that includes legal statutes, administrative penalties, and judicial verdicts dating back to the establishment of the People’s Republic of China. In this study, the two authors utilized the Lexis China database to conduct an advanced search of case files and verdict reports from January 2013 to December 2022, using ‘kidney transplantation surgery’ as the keyword. The collected data encompassed essential case information, clinical outcomes, and judgment results. For cases involving a second trial and retrial, the final judgment shall prevail. As the data is publicly accessible, institutional review board approval was not required.

A total of 304 cases were retrieved from the Lexis China database, and after excluding duplicates and irrelevant cases, 117 cases were included for analysis (Supplementary Fig. 1, Supplemental Digital Content 1, http://links.lww.com/JS9/A824). Kidney transplantation-related litigations exhibited a gradual increase over time, with a slight decline in the past 3 years, possibly linked to reduced organ transplantation during the COVID-19 pandemic (Fig. 1A)^5^. The average age of patients was 43.81 (±9.78) years, predominantly female (*N*=70, 59.83%). Patients (and families) served as plaintiffs in all the first trials. Except for one patient who served as a kidney donor, all remaining patients were kidney recipients, with more than 90% of them receiving living donor kidney transplantation (*N*=107, Table 1). Geographically, litigation cases are primarily concentrated in the economically advanced eastern regions of China, particularly Beijing and Guangdong (Supplementary Fig. 2, Supplemental Digital Content 1, http://links.lww.com/JS9/A824).

**Figure 1:**
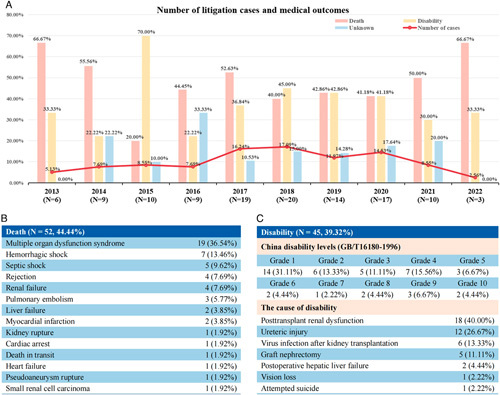
(A) Number of litigation cases and medical outcomes; (B) the cause of death; (C) disability grading and causes.

**Table 1:** Characteristics of kidney transplantation-related claims.

Number of cases	117
Age, year	
Median (IQR)	44 (39, 49)
Means (SD)	43.81 (9.78)
Sex, *N* (%)	
Male	47 (40.17)
Female	70 (59.83)
Plaintiff(s) identity, *N* (%)	
Patients (and families)	103 (88.03)
Hospital	14 (11.97)
Type of patient, *N* (%)	
Kidney donors	116 (99.15)
Kidney recipients	1 (0.085)
Surgery to litigation interval, year	
Median (IQR)	2.00 (1.00, 3.00)
Means (SD)	2.96 (2.70)
Time lag between litigation and legal verdict, year	
Median (IQR)	1.00 (0.00, 2.00)
Means (SD)	1.51 (1.25)
Time lag between litigation and legal verdict, *N* (%)	
≤1 year	76 (64.96)
>1 year	41 (35.04)
Settlement amount, USD	
Median (IQR)	97 284 (36 366, 140 881)
Means (SD)	108 824 (41 764)
Surgical procedure, *N* (%)	
Living donor kidney transplantation	107 (91.45)
Cadaveric kidney transplantation	7 (5.98)
Renal autotransplantation	3 (2.56)
Legal verdict on medical fault percentage, *N* (%)	
None	17 (14.53)
Mild	21 (17.95)
Equal	31 (26.50)
Predominant	38 (32.48)
All	10 (8.54)
Distribution of claimed breaches of duty, *N* (%)	
Negligence in preoperative informed consent	19 (16.24)
Negligence in intraoperative performance	37 (31.62)
Negligence in postoperative care	44 (37.61)
No liability for the medical provider	17 (14.53)
Litigation process, *N* (%)	
Case closed at first trial	78 (66.67)
Case closed at second trial or retrial	39 (33.33)

IQR, interquartile range; SD, standard deviation.

Continuous variables are reported as mean (SD), median (IQR); categorical variables, as frequencies (proportions).

The majority of litigation cases ended in the first trial (*N*=78, 66.67%). Patient mortality accounted for 44.44% of the cases (*N*=52), primarily due to multiple organ dysfunction syndrome and hemorrhagic shock (Fig. 1B). Among the 49 patients with adverse clinical outcomes resulting in disability, over 30% suffered complete work incapacity classified as level I disabilities. The leading causes of disability were posttransplant renal dysfunction and ureteral injury (Fig. 1C). Minimal cases (*N*=17, 14.53%) were determined to have no medical fault or negligible causal relationship with patient harm by third-party appraisal agencies and courts. Medical responsibility encompassed negligence in obtaining informed consent preoperatively (*N*=19, 16.24%), intraoperative errors (*N*=37, 31.62%), and postoperative care negligence (*N*=44, 37.61%). These findings emphasize the critical importance of precise intraoperative procedures and vigilant postoperative monitoring. Notably, a patient made a suicide attempt following surgery, underscoring the significance of attending to patients’ mental health. The mean final judgment in the aforementioned 117 legal cases amounted to 108 824 (±41 764) USD, significantly surpassing the costs of kidney transplant surgeries in China.

Although the doctor–patient relationship should be characterized by equality, patients often face vulnerability. In the realm of kidney transplantation, surgeons should communicate extensively with patients prior to surgery and assess perioperative risks diligently. Emphasizing intraoperative precision to minimize avoidable harm and maintaining postoperative vigilance for timely complication management is crucial. Surgeons should enhance their understanding of medical laws and exercise caution in their practice. In essence, reducing medical disputes will indirectly enhance healthcare quality and improve clinical outcomes for kidney transplant recipients.

## Ethical approval

As the data is publicly accessible, institutional review board approval was not required.

## Source of funding

None.

## Author contribution

Q.H.: conceptualization, data acquisition, and writing; Z.T: statistical analysis, creation of table and figure, and writing; L.Z.: review, editing, and supervision.

## Conflicts of interest disclosure

The authors declare no conflicts of interest.

## Research registration unique identifying number (UIN)

1. Name of the registry: not applicable.
2. Unique identifying number or registration ID: not applicable.
3. Hyperlink to your specific registration (must be publicly accessible and will be checked): not applicable.

## Guarantor

All authors.

## Data availability statement

Data are available from the corresponding author if the justification for the requirement is justified.

## Supplementary Material
