# Supplementary material for: Litigation on kidney transplantation: 10-year experience from China
Source: Int J Surg. 2023 Jul 31;109(11):3700–2. doi: 10.1097/JS9.0000000000000625 (PMC10651275; doi:10.1097/JS9.0000000000000625)
Supplement: Supplementary file 1 [file js9-109-3700-s001.docx]

**
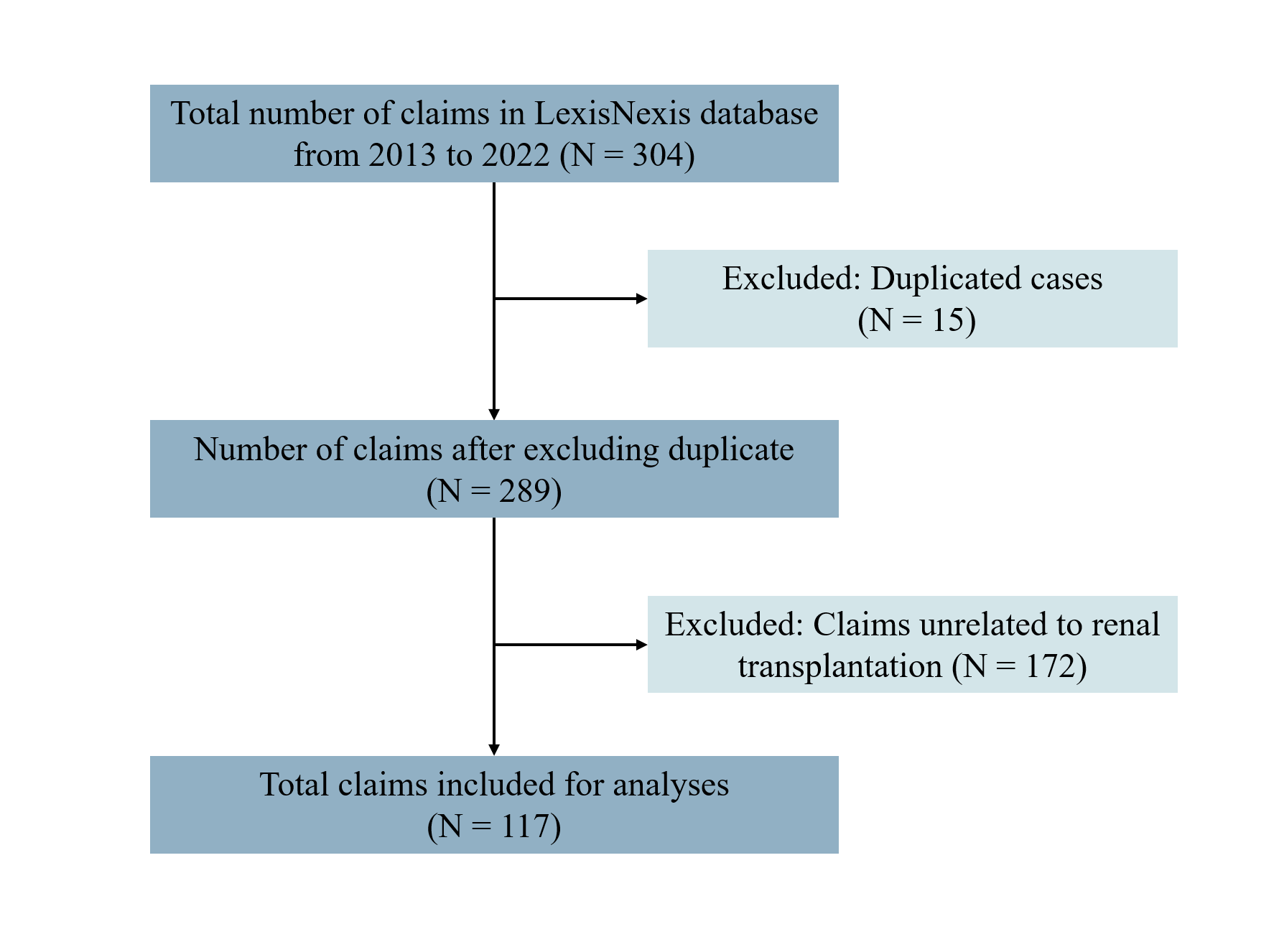
**

**Supplementary Figure 1** Flow chart of database analysis showing the exclusions and the total claims analyzed.


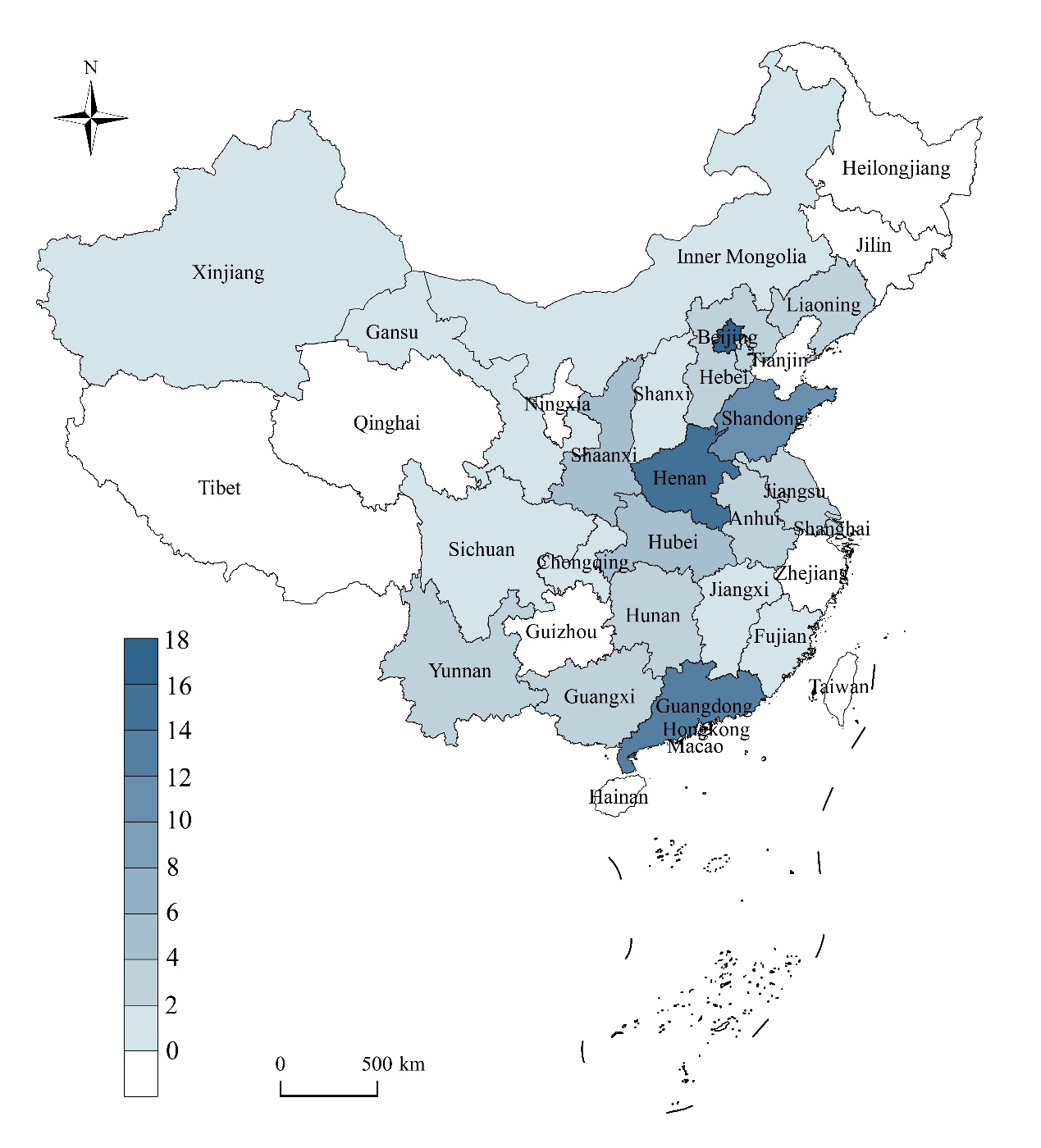


**Supplementary Figure 2** Provincial distribution of litigation on kidney transplantation.
